# MambaReID: Exploiting Vision Mamba for Multi-Modal Object Re-Identification

**DOI:** 10.3390/s24144639

**Published:** 2024-07-17

**Authors:** Ruijuan Zhang, Lizhong Xu, Song Yang, Li Wang

**Affiliations:** 1 School of Computer and Information, Hohai University, Nanjing 211106, China; zhangruijuan@hytc.edu.cn (R.Z.); hhu1979hhu@hhu.edu.cn (L.X.); 150207080003@hhu.edu.cn (S.Y.); 2 School of Mathematics and Statistics, Huaiyin Normal University, Huai’an 223300, China; 3 School of Computer and Software, Nanjing Vocational University of Industry Technology, Nanjing 210023, China

**Keywords:** multi-modal object ReID, VMamba, dense connection, consistent VMamba fusion, modal aggregation

## Abstract

Multi-modal object re-identification (ReID) is a challenging task that seeks to identify objects across different image modalities by leveraging their complementary information. Traditional CNN-based methods are constrained by limited receptive fields, whereas Transformer-based approaches are hindered by high computational demands and a lack of convolutional biases. To overcome these limitations, we propose a novel fusion framework named MambaReID, integrating the strengths of both architectures with the effective VMamba. Specifically, our MambaReID consists of three components: Three-Stage VMamba (TSV), Dense Mamba (DM), and Consistent VMamba Fusion (CVF). TSV efficiently captures global context information and local details with low computational complexity. DM enhances feature discriminability by fully integrating inter-modality information with shallow and deep features through dense connections. Additionally, with well-aligned multi-modal images, CVF provides more granular modal aggregation, thereby improving feature robustness. The MambaReID framework, with its innovative components, not only achieves superior performance in multi-modal object ReID tasks, but also does so with fewer parameters and lower computational costs. Our proposed MambaReID’s effectiveness is validated by extensive experiments conducted on three multi-modal object ReID benchmarks.

## 1. Introduction

Object Re-identification (ReID) aims to re-identify the same object across different camera views. Due to its wide range of applications, object ReID [1,2,3] has advanced significantly in recent years. In particular, traditional object ReID mainly focuses on extracting discriminative information from easily accessible RGB images. However, various complex imaging conditions like darkness, strong lighting, and low image resolution may severely affect the quality of RGB images [4]. The critical regions of objects become blurred [5], resulting in a loss of discriminative information. Fortunately, multi-modal object ReID [6,7,8] has shown significant potential in overcoming the challenges. By integrating complementary information from near-infrared (NIR), thermal infrared (TIR), and RGB images, multi-modal object ReID can provide more robust representations in complex scenes. Thus, it has attracted increasing attention in the past few years.

In order to aggregate heterogeneous information from different modalities, Li et al. [5] first propose a multi-modal vehicle ReID benchmark named RGBNT100, which contains RGB, NIR, and TIR images. Meanwhile, they propose a HAMNet to learn the robust representations with a heterogeneous coherence loss. Further, Zheng et al. [4] propose a PFNet to progressively fuse multi-modal features, along with the first multi-modal person ReID benchmark named RGBNT201. Wang et al. [9] employ three learning methods to enhance modality-specific knowledge with the IEEE framework. Then, Zheng et al. [6] introduce a DENet to tackle the modality-missing issue. With generative models, Guo et al. [10] present a GAFNet that fuses heterogeneous information. Meanwhile, with the generalization ability of Transformers [11], researchers begin to explore the potential of Transformers in multi-modal object ReID. Pan et al. [12] construct a PHT, utilizing a feature hybrid mechanism to balance information from different modalities. Through analyzing the modality of laziness, Jennifer et al. [13] provide a strong baseline UniCat. Further, Wang et al. [7] introduce a new token permutation mechanism designed to enhance the robustness of multi-modal object ReID. From the perspective of test-time training, Wang et al. [14] propose a HTT to explore the information existing in the test data. Recently, Zhang et al. [8] construct an EDITOR to select diverse features and minimize the influence of background noise. Although these methods achieve promising results, they still have some limitations.

For CNN-based methods, their performance is hindered by limited receptive fields, making them difficult to capture global information from heterogeneous modalities. As for Transformer-based methods, while they exhibit superior performance, the quadratic computational complexity [11] introduced by attention mechanisms is unacceptable. Additionally, the lack of convolutional inductive bias [15] in Transformer-based methods results in weaker perception of local details, leading to the neglect of certain information among different modalities. Thus, efficient integration of global and local information becomes crucial for multi-modal object ReID. However, existing methods may fail to exploit the complementary advantages of the above frameworks. On the one hand, the features extracted by existing methods lack sufficient robustness. On the other hand, the most effective current approaches, which employ highly complex Transformer models, are not efficient. Therefore, there is an urgent need for an efficient method capable of extracting robust features.

Recently, Mamba [16] has drawn significant attention [17] due to its superior scalability with State Space Models (SSM) [18]. Empowered by the Selection Mechanism (S6), Mamba surpasses alternative state-of-the-art architectures, like CNNs or Transformers, in managing long sequences with linear complexity. Furthermore, Mamba has been successfully applied to various computer vision tasks [19,20,21], such as image classification, object detection, and video understanding. To be specific, VMamba [19] integrates S6 with a four directions scanning mechanism, which can fully capture the global context information and local details. Drawing inspiration from VMamba’s exceptional performance in image classification tasks, we introduce a novel fusion framework named MambaReID, specifically designed for multi-modal object ReID. This innovative approach leverages the strengths of various modalities to enhance the re-identification process, aiming to significantly improve accuracy and efficiency in multi-modal object ReID scenarios.

Technically, our proposed MambaReID consists of three main components: Three-Stage VMamba (TSV), Dense Mamba (DM), and Consistent VMamba Fusion (CVF). More specifically, TSV is designed to extract robust multi-modal representations with four directions scanning mechanism. Instead of directly transferring VMamba, we observe that its final stage downsampling leads to substantial detail loss and computational redundancy. For tasks like multi-modal ReID, high-resolution feature maps provide more details for subsequent modality fusion. Hence, we opt to skip the final stage and adopt a last stride technique, as described in BoT [22]. This adaptation allows TSV to preserve richer details while reducing computational overhead, resulting in more robust outcomes compared to Transformers. Then, we introduce a DM into the TSV to further enhance the discriminative ability. Dense connections are crucial for fine-grained image classification tasks [23], as they offer semantic information at different levels, thereby enhancing the robustness of features. With the dense connections, DM can fully integrate the inter-modallity information with shallow and deep features. Different from previous dense connections, we only introduce that in the last stage of TSV with a small computational overhead. Hence, MambaReID can retrain more fine-grained details with less computational cost. Finally, to effectively integrate information from multiple modalities, we introduce the CVF. CVF incorporates a consistent loss function to align deep features across different modalities. This loss ensures that features from different modalities are well-aligned, facilitating effective fusion. Aligned features are then concatenated along the channel dimension and processed by the VMamba block for modality integration. This step enables simultaneous integration of information from multiple modalities at identical spatial positions, thereby enhancing the granularity of modal aggregation. Through our method, CVF ensures the effective utilization of well-aligned multi-modal images. Finally, with our proposed method, MambaReID can provide more robust multi-modal features for multi-modal ReID. Extensive experiments on three public benchmarks demonstrate that our MambaReID can achieve superior performance compared to most state-of-the-art methods.

In summary, our contributions are as follows:

- We introduce a novel fusion framework named MambaReID. This work marks the first attempt at integrating Mamba into multi-modal object ReID.
- We propose the Three-Stage VMamba (TSV) to extract robust multi-modal representations. TSV efficiently captures both global context information and local details, while maintaining low computational complexity.
- We introduce Dense Mamba (DM) to seamlessly integrate inter-modality information across shallow and deep features. Moreover, we present Consistent VMamba Fusion (CVF) to fuse deep features originating from diverse modalities. Leveraging well-aligned multi-modal images, CVF refines modal aggregation granularity, consequently enhancing feature discriminability.

## 2. Proposed Methodology

### 2.1. Overall Architecture of MambaReID

**Figure 1:**
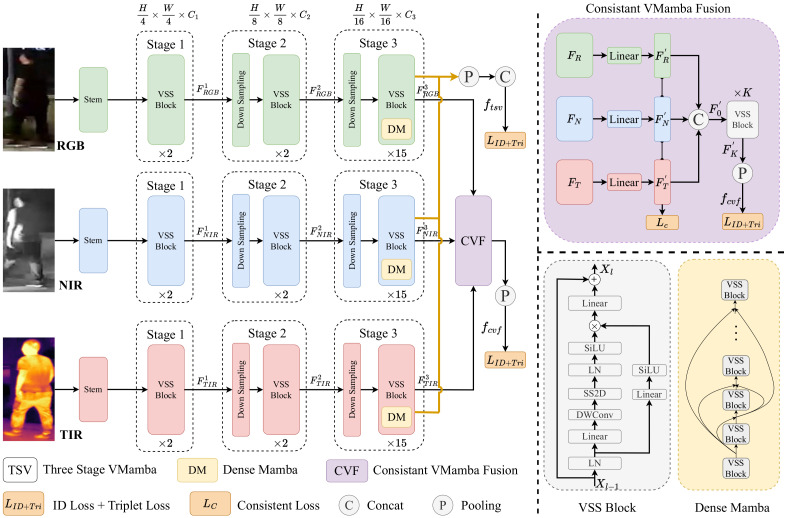
The overall architecture of our MambaReID. First, the images form RGB, NIR and TIR modalities are fed into the backbone TSV. With the lightweight TSV, we can extract robust multi-modal representations from different modalities. Then, in the last stage of TSV, DM is utilized to further intergrate the information from both shallow and deep features. Finally, with the well-aligned multi-modal images, CVF is employed to enhance the modal aggregation granularity at the same spatial positions. Thanks to the proposed modules, our MambaReID generates more discriminative multi-modal information with low computational complexity.

As shown in Figure 1, our MambaReID consists of three main components: Three-Stage VMamba (TSV), Dense Mamba (DM), and Consistent VMamba Fusion (CVF). We employ the TSV as the backbone. It is designed to extract robust single-modal representations from RGB, NIR, and TIR modalities. DM is introduced at the final stage of TSV to further improve network discriminative capability. Additionally, with the well-aligned multi-modal images, CVF is employed to enhance the modal aggregation granularity. Detailed descriptions of our proposed modules are provided in the following sections.

### 2.2. Preliminary

**State Space Models (SSMs).** SSMs [24,25] have been widely employed across diverse sequential data modeling tasks. Inspired by continuous systems, SSMs adeptly capture the dynamic patterns inherent in input sequences. To be specific, SSMs can map the input $x\left(t\right)\in R^{L}$ to the output $y\left(t\right)\in R^{L}$, which is expressed as:

(1) $$\dot{h}\left(t\right)=Ah\left(t\right)+Bx\left(t\right),$$

(2) y(t)=Ch(t)+Dx(t),

where $A\in C^{N\times N}$, $B\in C^{N}$ and $C\in C^{N}$ are the model parameters, while the D∈C is the residual term. Here, *N* is the dimension of the hidden state h(t). The above equations can easily model the continuous input. However, in the context of a discrete input like image or text, SSM needs to be discretized with the zero-order hold (ZOH) method. To specify, where $\Delta \in R^{D}$ represents the predefined timescale parameter that maps the continuous parameters *A* and *B* into a discrete space, the discretization process is described as:

(3) $$\bar{A}=e^{\Delta A},$$

(4) $$\bar{B}=\left(e^{\Delta A}-I\right)A^{-1}\Delta B,$$

(5) $$\bar{C}=C,$$

where $B,C\in R^{D\times N}$ are the discretized matrices. After discretization, the SSM is calculated as follows:

(6) $$\;h_{k}=\bar{A}h_{k-1}+\bar{B}x_{k}$$

(7) $$y_{k}=\bar{C}h_{k}+\bar{D}x_{k}.$$

Finally, with the discretized SSM, we can model the discrete image with linear complexity.

**Selective Scan Mechanism.** SSM can be utilized for efficient sequence modeling. However, the conventional SSM may fail to capture the complex patterns in various input sequences. Without a data-dependent structure, SSM lacks the ability to focus on or ignore specific information. To solve this issue, Gu et al. [16] present a Selective Scan mechanism for SSM (S6), where the matrices $B\in R^{L\times N}$, $C\in R^{L\times N}$, and $\Delta \in R^{L\times D}$ are generated by the input data $x\in R^{L\times D}$. This enables S6 to fully perceive the contextual information of the input, rendering it more flexible and efficient.

**Figure 2:**
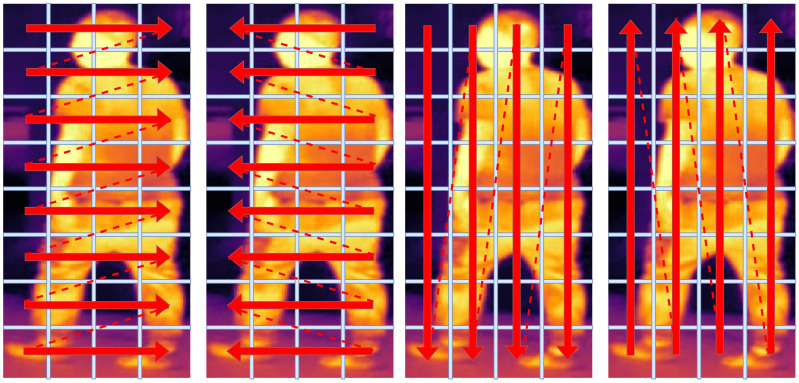
The visualization of 2D Selective Scan (SS2D) mechanism based on TIR images. From left to right, the SS2D scans the input image in four directions for comprehensive information perception. Different directions of the red arrows represent different scanning directions.

**2D Selective Scan.** As shown in the first image of Figure 2, the S6 usually scans the input sequence in one direction. However, in the context of visual tasks, the sequences we encounter often consist of non-causal image data. Thus, the unidirectional scanning method employed in S6 is deemed impractical. Within single direction scanning, the current image patch can only perceive the information from the previous patches, instead of the local information from different directions. To address this issue, VMamba [19] introduces a 2D Selective Scan (SS2D) mechanism. To be specific, SS2D scans the input sequence in four directions: left-to-right, right-to-left, top-to-bottom, and bottom-to-top. As shown in Figure 2, the patch in a specific region can perceive the information from adjacent patches in different directions, which can enhance the feature discriminability with contextual information. Thus, in our MambaReID, we employ the SS2D to extract robust multi-modal representations. Specifically, we only need to unfold the images in different sequences. After scanning, we restore them according to their original relative positions, and then sum the scanning results from the four directions at the same position.

### 2.3. Three-Stage VMamba

To fully exploit the discriminative information with low computational complexity, we first introduce the VMamba as our backbone. Generally, the VMamba is composed of four stages with three downsampling operations. However, the downsampling of the last stage in VMamba leads to a significant loss of detailed information. Moreover, the final stage introduces redundant computational costs. Therefore, we eliminate the last downsampling like BoT [22] to preserve richer details. Further, we directly remove the final stage to achieve more efficient modeling. Thus, we propose the Three-Stage VMamba (TSV) as our backbone, as shown in Figure 1.

To be specific, we denote the input multi-modal images as $X=\{X_{\mathrm{RGB}},X_{\mathrm{NIR}},X_{\mathrm{TIR}}\in R^{H\times W\times C}$}, where *H*, *W*, and *C* denote the height, width, and channel of the images, respectively. For illustrative purposes and without loss of generality, we consider the RGB modality as a representative example. The RGB images $X_{\mathrm{RGB}}$ are first fed into the Stem block with a convolutional layer to extract the initial features $F_{\mathrm{RGB}}\in R^{\frac{H}{4}\times \frac{W}{4}\times C_{1}}$. Then, $F_{\mathrm{RGB}}$ are fed into the VSS block to integrate the global and local information. Technically, the VSS block consists of LayerNorm (LN) [26], Linear transformation, Depthwise Convolution (DWConv) [27], SS2D and SiLU [28] activation functions. As shown in the right corner of Figure 1, the VSS block can be formulated as:

(8) $$\Psi^{i}\left(X\right)={\mathrm{Linear}}^{i}\left(\mathrm{LN}\left(X\right)\right),i\in \left\{1,2\right\},$$

(9) $$\Omega \left(X\right)=\mathrm{LN}(\mathrm{SS}2D\left(\mathrm{DWConv}\left(\Psi^{1}\left(X\right)\right)\right)),$$

(10) $$\mathrm{VSS}\left(X\right)=\mathrm{Linear}(\mathrm{SiLU}\left(\Omega \left(X\right)\right)\times \mathrm{SiLU}\left(\Psi^{2}\left(X\right)\right)).$$

Then, with the residual connection, we fed the output of the current VSS block into the next VSS block as follows:

(11) $$X_{l}=X_{l-1}+{\mathrm{VSS}}_{l}\left(X_{l-1}\right),$$

where $X_{l}$ denotes the output of the *l*-th VSS block. Thus, after the first stage of TSV, we can obtain the features $F_{\mathrm{RGB}}^{1}\in R^{\frac{H}{4}\times \frac{W}{4}\times C_{1}}$. Then, the features $F_{\mathrm{RGB}}^{1}$ are fed into the next stage for further processing. Similarly, we can obtain the features $F_{\mathrm{RGB}}^{2}\in R^{\frac{H}{8}\times \frac{W}{8}\times C_{2}}$. Finally, with the last stage of TSV, we obtain the features $F_{\mathrm{RGB}}^{3}\in R^{\frac{H}{16}\times \frac{W}{16}\times C_{3}}$. Similar to the RGB modality, we can also extract the features $F_{\mathrm{NIR}}^{3}$ and $F_{\mathrm{TIR}}^{3}$. Then, we employ the pooling (P) on $F_{\mathrm{RGB}}^{3}$, $F_{\mathrm{NIR}}^{3}$ and $F_{\mathrm{TIR}}^{3}$, respectively. Finally, we concatenate the pooled features to obtain the multi-modal features $f_{tsv}$ as follows:

(12) $$f_{tsv}=\left[P\left(F_{\mathrm{RGB}}^{3}\right),P\left(F_{\mathrm{NIR}}^{3}\right),P\left(F_{\mathrm{TIR}}^{3}\right)\right],$$

where [·] is the concatenation operation. After applying the loss supervision on $f_{tsv}$, we can extract the robust multi-modal representations with TSV.

### 2.4. Dense Mamba

Dense connections [29] have been extensively utilized in various computer vision tasks. They can effectively enhance the feature discriminability by fully integrating the information with shallow and deep features. However, directly introducing dense connections into the TSV will lead to a significant increase in complexity. Additionally, the effectiveness of dense connections in VMamba remains unclear. Therefore, we explore the potential of dense connections in VMamba and introduce the Dense Mamba (DM) to the last stage of TSV. As shown in the right corner of Figure 1, we simplify the dense connections by directly adding the previous features to the current features. To be specific, the DM can be formulated as:

(13) $$X_{l}={\bar{X}}_{l-1}+{\mathrm{VSS}}_{l}\left({\bar{X}}_{l-1}\right),$$

(14) $${\bar{X}}_{l}=\frac{1}{l}\sum_{i=1}^{l}X_{i},$$

where *l* denotes the *l*-th VSS block in the last stage of TSV. With the simple dense connections, we can fully integrate the information from different levels within a single modality. Meanwhile, the computational overhead is extremely low.

### 2.5. Consistent VMamba Fusion

To fully exploit the complementary information from different modalities, we introduce the Consistent VMamba Fusion (CVF). With well-aligned multi-modal images, we employ consistent loss to integrate the deep features across modalities at the same spatial positions. Furthermore, we concatenate the aligned features along the channel dimension and feed them into the VSS blocks to achieve modality fusion. The detailed process of CVF is illustrated in the right corner of Figure 1. We first directly copy the features $F_{\mathrm{RGB}}^{3}$, $F_{\mathrm{NIR}}^{3}$ and $F_{\mathrm{TIR}}^{3}$ to obtain $F_{R},F_{N}$ and $F_{T}$, respectively. Then, we utilize a linear transformation to reduce the channel dimension of $F_{R},F_{N}$ and $F_{T}$ to $F_{R}^{\prime },F_{N}^{\prime }$ and $F_{T}^{\prime }\in R^{\frac{H}{16}\times \frac{W}{16}\times \frac{C_{3}}{3}}$:

(15) $$F_{R}^{\prime }=\mathrm{Linear}\left(F_{R}\right),$$

(16) $$F_{N}^{\prime }=\mathrm{Linear}\left(F_{N}\right),$$

(17) $$F_{T}^{\prime }=\mathrm{Linear}\left(F_{T}\right).$$

Then, we employ the consistent loss to align the features $F_{R}^{\prime },F_{N}^{\prime }$ and $F_{T}^{\prime }$ with the following equations:

(18) $$L_{R\Leftrightarrow N}=\left|\right|F_{R}^{\prime }-F_{N}^{\prime }{\left|\right|}_{2}^{2},$$

(19) $$L_{R\Leftrightarrow T}=\left|\right|F_{R}^{\prime }-F_{T}^{\prime }{\left|\right|}_{2}^{2},$$

(20) $$L_{N\Leftrightarrow T}=\left|\right|F_{N}^{\prime }-F_{T}^{\prime }{\left|\right|}_{2}^{2}.$$

We end up with the consistency constraint loss $L_{C}$, which can be represented as:

(21) $$L_{C}=\frac{1}{N_{p}}\left(L_{R\Leftrightarrow N}+L_{R\Leftrightarrow T}+L_{N\Leftrightarrow T}\right),$$

where $N_{p}$ is the number of patches. After obtaining the aligned features, we concatenate them along the channel dimension to obtain the multi-modal features $F_{0}^{\prime }\in R^{\frac{H}{16}\times \frac{W}{16}\times C_{3}}$ as follows:

(22) $$F_{0}^{\prime }=\left[F_{R}^{\prime },F_{N}^{\prime },F_{T}^{\prime }\right],$$

Then, we feed the $F_{0}^{\prime }$ into the stacked *K* layers of VSS blocks:

(23) $$F_{l}^{\prime }=F_{l-1}^{\prime }+{\mathrm{VSS}}_{l}\left(F_{l-1}^{\prime }\right),l\in \left\{1,...,K\right\},$$

where $F_{l}^{\prime }$ denotes the output of the *l*-th VSS block in the CVF.

(24) $$f_{cvf}=P\left(F_{K}^{\prime }\right),$$

Finally, we pool the output of the last VSS block to obtain the features $f_{cvf}\in R^{C_{3}}$. Subsequently, the features $f_{cvf}$ are sent for loss supervision. With the fully integrated multi-modal features, we can obtain more discriminative representations.

### 2.6. Objective Function

As depicted in Figure 1, our objective function comprises three parts: losses for the TSV backbone, CVF, and the consistency constraint loss $L_{C}$. Both the backbone and CVF are supervised by the label-smoothing cross-entropy ID loss $L_{\mathrm{ID}}$ [30] and triplet loss $L_{\mathrm{Triplet}}$ [31]:

(25) $$L_{\mathrm{TSV}}=L_{\mathrm{ID}}\left(f_{tsv}\right)+L_{\mathrm{Triplet}}\left(f_{tsv}\right),$$

(26) $$L_{\mathrm{CVF}}=L_{\mathrm{ID}}\left(f_{cvf}\right)+L_{\mathrm{Triplet}}\left(f_{cvf}\right).$$

The overall objective function is defined as:

(27) $$L=L_{\mathrm{TSV}}+L_{\mathrm{CVF}}+\lambda L_{C},$$

where λ is the hyperparameter used to balance $L_{C}$. By minimizing the L, our MambaReID can generate more discriminative multi-modal features with low computational complexity.

## 3. Experiments

### 3.1. Dataset and Experimental Setup

We utilize three multi-modal object ReID benchmarks to evaluate the performance of our proposed MambaReID. Particularly, RGBNT201 [4] is the inaugural multi-modal dataset for person re-identification, which includes RGB, NIR, and TIR modalities. RGBNT100 [5] is a large-scale multi-modal vehicle ReID dataset, while MSVR310 [32] is a smaller-scale multi-modal vehicle ReID dataset featuring complex visual scenes. Regarding the evaluation metrics, we align with prior studies and adopt mean Average Precision (mAP) and Cumulative Matching Characteristics (CMC) at Rank-K (K=1,5,10). Additionally, we report the trainable parameters and FLOPs for complexity analysis.

### 3.2. Implementation Details

We leverage pre-trained VMamba [19], sourced from the ImageNet classification dataset [33], as the backbone of our architecture. The images of RGBNT201 were resized to 256 × 128 and the images of RGBNT100 and MSVR310 datasets were resized to 128 × 256 during data processing. In the training process, we enhance the robustness of our model by applying data augmentation techniques such as random horizontal flipping, cropping, and erasing [34]. The training process is managed with a mini-batch size of 64, consisting of eight randomly selected identities, each providing eight images. Optimization of our model is achieved through the use of Stochastic Gradient Descent (SGD) with a momentum of 0.9 and a weight decay of 0.0001. The learning rate is initially set to 0.01 and a cosine decay warm-up strategy is applied. The hyperparameter λ in L is set to 1. For the CVF, we set the K to 1. During testing, we concatenate the features $f_{tsv}$ and $f_{cvf}$ to obtain the final multi-modal features for retrieval. Specifically, each query consists of three paired images: RGB, NIR, and TIR. The model takes these three modalities as input and extracts the final retrieval vector according to the network structure, which serves as the feature of the query. A similar process is applied to each triplet in the gallery. Finally, by ranking the similarity between the features of the query and those in the gallery, the model determines whether the object is correctly matched. The proposed method is implemented using the PyTorch framework and experiments are conducted on a single NVIDIA A100 GPU (NVIDIA, Santa Clara, CA, USA).

### 3.3. Comparisons With State-of-the-Art Methods

**Multi-modal Person ReID.** As reported in Table 1a, we conduct comprehensive comparisons with dominant single-modal and multi-modal approaches on RGBNT201. Typically, single-modal approaches perform poorly due to the lack of specialized designs for multi-modal fusion. Among single-modal methods, PCB demonstrates a notable mAP of 32.8%. For multi-modal methods, CNN-based frameworks demonstrate inferior performance compared to Transformer-based ones due to their limited receptive fields. With the strong generalization ability of Transformers, HTT [14], TOP-ReID [7] and EDITOR [8] achieve superior performance. Specifically, TOP-ReID (A) achieves a mAP of 72.3%, surpassing HTT by 1.2%. However, the high computational complexity of Transformers is unacceptable. Based on VMamba, with only 18.32% of the TOP-ReID’s parameters, our MambaReID achieves a mAP of 72.2%. Additionally, our MambaReID outperforms most CNN-based and Transformer-based methods across various settings (A/B settings), thereby validating the efficacy of our approach.

**Multi-modal Vehicle ReID.** As depicted in Table 1b, we compare our MambaReID with mainstream methods on RGBNT100 and MSVR310. In single-modal methods, BoT [22] achieves a mAP of 78.0% on RGBNT100. Transformer-based method TransReID [45] underperforms CNN-based methods due to the lack of convolutional inductive bias. Especially on the small-scale dataset MSVR310, TransReID achieves a mAP of 18.4%, which is 10.5% lower than AGW [1]. However, in multi-modal methods, Transformer-based methods like TOP-ReID [7] and EDITOR [8] exhibit superior performance in integrating multi-modal information. Focusing on mitigating the influence of irrelevant background, EDITOR achieves a mAP of 82.1% on RGBNT100. With only 50.2% trainable parameters of EDITOR, our MambaReID achieves a competitive mAP of 78.6% on RGBNT100. Additionally, on MSVR310, our MambaReID (B) achieves a mAP of 46.1%, surpassing EDITOR (B) by 7.1%. Overall, these results fully demonstrate the effectiveness of our proposed method.

### 3.4. Ablation Study

**Effect of different components.** In Table 2, we conduct ablation studies to evaluate the effectiveness of different components on RGBNT201. Model A is the baseline model with only TSV. With the introduction of CVF, model B achieves a mAP of 68.03%, surpassing model A by 4.22%. Through introducing DM, model C achieves a mAP of 68.07%, surpassing model A by 4.26%. Finally, with both DM and CVF, our MambaReID achieves a mAP of 72.20%, surpassing model B and model C by 4.17% and 4.13%, respectively. As for complexity, model B and model C only introduce a small computational overhead. Especially for model C, the computational overhead is negligible. These results fully authenticate the effectiveness of our proposed components.

**Parameter analysis.** In Table 3, we compare the trainable parameters of our MambaReID with mainstream methods on RGBNT100. Compared to TOP-ReID [7] and EDITOR [8], our MambaReID achieves a competitive mAP of 78.6% on RGBNT100 with only 59.47 M parameters. Additionally, our MambaReID outperforms most CNN-based methods with a similar number of parameters. This clearly illustrates the efficiency and effectiveness of our proposed approach.

**Effect of different backbones.** In Table 4, we compare the performance of different backbones on RGBNT201. ViT achieves a mAP of 63.18%, while directly using VMamba achieves a mAP of 55.98%. This result indicates that the final stage downsampling of VMamba leads to substantial detail loss. With the last stride trick in BoT [22], VMamba ^‡^ achieves a mAP of 58.47%. However, the high computational complexity of VMamba ^‡^ is unacceptable. Thus, we directly drop the final stage of VMamba and adopt a last stride technique, resulting in a mAP of 63.81%. With only 63.96% of the parameters and 79.22% FLOPs of ViT, our TSV achieves a mAP of 63.81%, surpassing ViT by 0.63%. Finally, we employ the TSV as our backbone in subsequent experiments. These outcomes affirm the efficacy of our proposed backbone structure.

**Effect of different depths of CVF.** In Table 5, we evaluate the performance of different depths of CVF on RGBNT201. With the increase in depth, the performance of CVF gradually decreases. This result indicates that a deeper CVF may introduce more irrelevant information, leading to performance degradation. Hence, we opt to use a CVF with a depth of 1 in the other experiments.

**Effect of different DMs.** In Table 6, we evaluate the performance of different DMs on RGBNT201. Specifically, “Last” refers to whether the output of TSV corresponds to the output of the last block or the sum of all blocks in the last stage. “Freq” refers to the frequency of dense connection is introduced in the last stage. When “Freq” is set to 2, the dense connections are introduced every two blocks in the last stage. Comparing model A and model B, integrating the output of all blocks in the last stage can introduce more fine-grained details. Additionally, with the “Freq” set to 2, model C achieves a mAP of 66.04%, surpassing model A by 2.53%. Finally, model D achieves the best performance with a mAP of 68.07%.

### 3.5. Visualization Analysis

**Figure 3:**
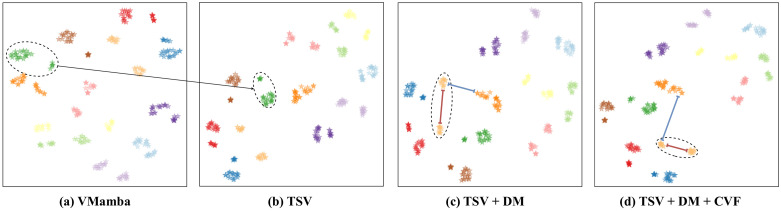
Feature distributions with t-SNE [48]. Different colors represent different IDs.

In Figure 3, we visualize the feature distributions of different modules on RGBNT201. In Figure 3a, the original VMamba’s extracted features for the same ID are widely dispersed, resulting in poor discrimination. Comparing Figure 3a,b, the introduction of TSV leads to tighter feature grouping within IDs and increased dispersion between different IDs. Additionally, the inclusion of DM results in a more compact feature distribution in Figure 3c. Comparing Figure 3d with Figure 3c, the introduction of CVF increases the spacing between indistinguishable IDs while reducing intra-ID spacing. These visualizations provide strong evidence of the efficacy and superiority of our modules.

## 4. Conclusions

In this study, we introduce a novel fusion framework, MambaReID, designed for multi-modal object ReID. We are the first to explore the potential of Mamba in multi-modal object ReID, and we find that its final stage critically disrupts the ReID features. Thus, MambaReID utilizes a Three-Stage VMamba (TSV) architecture to derive robust multi-modal representations that are rich in detail yet require lower computational resources. To enhance its discriminative power, we incorporate a Dense Mamba (DM) module within the TSV to fully exploit various levels of semantic features. Furthermore, leveraging well-aligned multi-modal images, we implement a Consistent VMamba Fusion (CVF) technique aimed at refining the granularity of modal integration. Comprehensive testing across three public benchmarks validates the superior performance and efficiency of our framework.

## Figures and Tables

**Table 1:** Comparative analysis of three multi-modal object ReID benchmarks, with the top and second-best performances highlighted in bold and underlined, respectively. The symbol * represents the Transformer-based methods, while † denotes the Mamba-based methods. For the rest of the methods, they are CNN-based methods. The A and B denote the different settings from TOP-ReID [7].

(a) Comparison on RGBNT201						
Methods			RGBNT201			
Modality	Venue	Model	mAP	R-1	R-5	R-10
Single	ICCV’17	MUDeep [35]	23.8	19.7	33.1	44.3
	CVPR’18	HACNN [36]	21.3	19.0	34.1	42.8
	CVPR’18	MLFN [37]	26.1	24.2	35.9	44.1
	ECCV’18	PCB [38]	32.8	28.1	37.4	46.9
	ICCV’19	OSNet [39]	25.4	22.3	35.1	44.7
	ICCV’21	CAL [40]	27.6	24.3	36.5	45.7
Multi	AAAI’20	HAMNet [5]	27.7	26.3	41.5	51.7
	AAAI’21	PFNet [4]	38.5	38.9	52.0	58.4
	AAAI’22	IEEE [9]	49.5	48.4	59.1	65.6
	ArXiv’23	DENet [6]	42.4	42.2	55.3	64.5
	NeurIPSW’23	UniCat * [13]	57.0	55.7	-	-
	AAAI’24	HTT * [14]	71.1	73.4	83.1	87.3
	AAAI’24	TOP-ReID (A) * [7]	**72.3**	**76.6**	**84.7**	**89.4**
	AAAI’24	TOP-ReID (B) * [7]	64.6	64.6	77.4	82.4
	CVPR’24	EDITOR (A) * [8]	66.5	68.3	81.1	88.2
	CVPR’24	EDITOR (B) * [8]	65.7	68.8	82.5	89.1
	Ours	MambaReID ^†^ (A)	72.2	76.0	84.0	89.0
	Ours	MambaReID ^†^ (B)	65.1	67.4	78.4	84.4
**(b) Comparison on RGBNT100 and MSVR310**						
**Methods**			**RGBNT100**		**MSVR310**	
**Modality**	**Venue**	**Model**	**mAP**	**R-1**	**mAP**	**R-1**
Single	ECCV’18	PCB [38]	57.2	83.5	23.2	42.9
	ACM MM’18	MGN [41]	58.1	83.1	26.2	44.3
	ICCV’19	DMML [42]	58.5	82.0	19.1	31.1
	CVPRW’19	BoT [22]	78.0	95.1	23.5	38.4
	ICCV’19	OSNet [39]	75.0	95.6	28.7	44.8
	CVPR’20	Circle Loss [43]	59.4	81.7	22.7	34.2
	ICCV’21	HRCN [44]	67.1	91.8	23.4	44.2
	TPAMI’21	AGW [1]	73.1	92.7	28.9	46.9
	ICCV’21	TransReID * [45]	75.6	92.9	18.4	29.6
Multi	AAAI’20	HAMNet [5]	74.5	93.3	27.1	42.3
	AAAI’21	PFNet [4]	68.1	94.1	23.5	37.4
	ICSP’22	GAFNet [10]	74.4	93.4	-	-
	Inform Fusion’22	CCNet [32]	77.2	96.3	36.4	55.2
	ArXiv’23	GraFT * [46]	76.6	94.3	-	-
	TITS’23	GPFNet [47]	75.0	94.5	-	-
	Sensors’23	PHT * [12]	79.9	92.7	-	-
	NeruIPSW’23	UniCat * [13]	79.4	96.2	-	-
	AAAI’24	HTT * [14]	75.7	92.6	-	-
	AAAI’24	TOP-ReID (A) * [7]	73.7	92.2	30.2	33.7
	AAAI’24	TOP-ReID (B) * [7]	81.2	96.4	35.9	44.6
	CVPR’24	EDITOR (A) * [8]	79.8	93.9	35.8	43.1
	CVPR’24	EDITOR (B) * [8]	**82.1**	**96.4**	39.0	49.3
	Ours	MambaReID ^†^ (A)	76.6	93.4	35.8	46.0
	Ours	MambaReID ^†^ (B)	78.6	94.3	**46.1**	**59.4**

**Table 2:** Comparative performance of different components.

	Module		Complexity		RGBNT201			
	DM	CVF	Params (M)	FLOPs (G)	mAP	R-1	R-5	R-10
A	✕	✕	55.67	26.99	63.81	67.57	77.97	83.77
B	✕	✓	59.47	27.56	68.03	70.96	79.88	84.69
C	✓	✕	55.67	26.99	68.07	72.38	82.70	86.96
D	✓	✓	59.47	27.56	72.20	75.96	83.97	89.00

**Table 3:** Comparative number of parameters for different methods. The symbol * represents the Transformer-based methods, while † denotes the Mamba-based methods. For the rest of the methods, they are CNN-based methods.

Methods		Params	RGBNT100	
Venue	Model	M	mAP	Rank-1
ECCV’18	PCB [38]	72.33	57.2	83.5
ICCV’19	OSNet [39]	7.02	75.0	95.6
AAAI’20	HAMNet [5]	78.00	74.5	93.3
Inform Fusion’22	CCNet [32]	74.60	77.2	96.3
ICSP’22	GAFNet [10]	130.00	74.4	93.4
ICCV’21	TransReID * [45]	278.23	75.6	92.9
NeruIPSW’23	UniCat * [13]	259.02	79.4	96.2
ArXiv’23	GraFT * [46]	101.00	76.6	94.3
AAAI’24	TOP-ReID * [7]	324.53	81.2	96.4
CVPR’24	EDITOR * [8]	118.55	82.1	96.4
Ours	MambaReID ^†^	59.47	78.6	94.3

**Table 4:** Performance comparison with different baselines. The symbol ‡ denotes the backbone’s last stride set to 1.

Model	Params	FLOPs	RGBNT201			
Backbone	M	G	mAP	Rank-1	Rank-5	Rank-10
ViT	87.04	34.07	63.18	64.58	77.91	84.56
VMamba	88.06	30.09	55.98	57.85	71.60	78.65
VMamba ^‡^	88.06	39.38	58.47	60.92	74.04	80.72
TSV	55.67	26.99	63.81	67.57	77.97	83.77

**Table 5:** Comparison of different depths of CVF.

CVF	Params	FLOPs	RGBNT201			
Depth	M	G	mAP	Rank-1	Rank-5	Rank-10
1	59.47	27.56	68.03	70.96	79.88	84.69
2	62.75	28.08	66.11	67.89	78.04	83.74
3	66.02	28.60	64.83	66.96	77.98	84.03
4	69.30	29.12	63.01	65.72	76.52	82.33
5	72.58	29.64	65.07	68.18	78.18	83.82
6	75.85	30.16	62.42	64.58	75.01	80.49

**Table 6:** Comparison of different DM settings.

	DM		Params	FLOPs	RGBNT201			
	Last	Freq	M	G	mAP	R-1	R-5	R-10
A	✓	1	55.67	26.99	63.51	68.03	78.94	84.63
B	✕	1	55.67	26.99	66.63	70.98	81.35	86.23
C	✓	2	55.67	26.99	66.04	70.51	81.02	86.18
D	✕	2	55.67	26.99	68.07	72.38	82.70	86.96

## Data Availability

The data presented in this study are available on request from the corresponding author.
